# Re-Examining the Association between Vitamin D and Childhood Caries

**DOI:** 10.1371/journal.pone.0143769

**Published:** 2015-12-21

**Authors:** Tom Dudding, Steve J. Thomas, Karen Duncan, Debbie A. Lawlor, Nicholas J. Timpson

**Affiliations:** 1 MRC Integrative Epidemiology Unit, School of Community and Social Medicine, University of Bristol, Bristol, United Kingdom; 2 School of Oral and Dental Science, Bristol University, Bristol, United Kingdom; UNC School of Dentistry, University of North Carolina-Chapel Hill, UNITED STATES

## Abstract

**Background:**

Previous studies have reported an inverse association between vitamin D and childhood dental caries, but whether this is causal is unclear.

**Objective:**

To determine the causal effect of circulating 25-hydroxyvitamin D concentration on dental caries experience, early caries onset and the requirement for a dental general anesthetic.

**Design:**

A Mendelian randomization study was undertaken, using genetic variants known to be associated with circulating 25-hydroxyvitamin D concentrations in 5,545 European origin children from the South West of England. Data on caries and related characteristics were obtained from parental and child completed questionnaires between 38 and 91 months and clinical assessments in a random 10% sample at 31, 44 and 61 months.

**Results:**

In multivariable confounder adjusted analyses no strong evidence for an association of 25-hydroxyvitamin D with caries experience or severity was found but there was evidence for an association with early caries onset, or having a general anesthetic for dental problems. In Mendelian randomization analysis the odds ratio for caries experience per 10 nmol/L increase in 25-hydroxyvitamin D was 0.93 (95% confidence interval: 0.83, 1.05; *P* = 0.26) and the odds ratio for dental general anaesthetic per 10 nmol/L increase in 25-hydroxyvitamin D was 0.96 (95% confidence interval: 0.75, 1.22; *P* = 0.72).

**Conclusions:**

This Mendelian randomization study provides little evidence to support an inverse causal effect of 25-hydroxyvitamin D on dental caries. However, the estimates are imprecise and a larger study is required to refine these analyses.

## Introduction

Dental caries is the localized destruction of susceptible tooth tissues by acidic by-products from bacterial fermentation of dietary products[1]. In most high income countries caries in primary teeth is decreasing, but it is still common. 31% of 5 year olds in the UK experienced decay in 2013[2], and similar rates exist in other European and North American countries[3, 4]. There are approximately 30,000 hospital admissions a year in the UK for dental related issues amongst children, with the majority of these being for treatment under general anesthetic (GA)[5]. Severe early childhood caries (S-ECC), an extreme presentation of childhood caries, is the most common reason for dental GA[5]. Although the risks of GA are low, there is substantial financial costs to the NHS and morbidity to the patient[6].

The role of vitamin D in primary caries has been debated and two mechanisms by which vitamin D might influence caries have been suggested. Vitamin D is likely to act through the vitamin D receptor and polymorphisms within this gene have been associated with dental caries[7]. Low vitamin D may facilitate topical demineralizing of teeth, in a similar way to its known action on bone, via reduced concentrations of calcium and phosphate ions. Vitamin D may also modulate caries via immunological factors such as cathelicidins[8, 9]. Vitamin D is recorded in blood by measuring 25(OH)D a precursor to the active form (1α, 25 hydroxyvitamin D).

Epidemiological and intervention studies investigating this relationship have been of variable quality and reported contradictory findings[10–13]. An umbrella review and meta-analysis of vitamin D and its health benefits, highlighted vitamin D supplementation of children as an effective way to reduce primary caries (relative risk 0.53 (95% confidence interval (CI): 0.43, 0.63)[14]. However, the author of the original review, that forms the majority of this evidence, described the findings as having low certainty and high sources of bias since few were randomized studies and most did not compare baseline characteristics or assess outcomes blind to interventions[15].

Mendelian randomization (MR) uses genetic variants known to be reliably associated with a risk factor of interest to derive estimates of the causal effect of that risk factor on health outcomes[16, 17]. Single nucleotide polymorphisms (SNPs) in the genes *CYP2R1*, *DHCR7* and *GC* (rs10741657, rs7944926 and rs2282679) are associated with 25(OH)D concentrations [18, 19]. CYP2R1 and DHCR7 encode proteins involved in the 25(OH)D synthetic pathway and GC encodes a 25(OH)D transport protein. The three SNPs are not thought to effect dental caries directly, other than via 25(OH)D. These SNPs have previously been used in MR studies to examine the causal effect of vitamin D on all-cause mortality, cardiovascular and cancer mortality and obesity in adults[20, 21]. Furthermore, the difference in mean 25(OH)D between the minor and major homozygotes for the *GC* variant has been shown to be of a similar magnitude to that of supplementation[19].

This study aims to investigate the relationships between 25(OH)D and childhood caries experience, onset and severity, as well as dental GA experience. Causal effects will be estimated by conducting MR using a genetic instrument to proxy the effect of 25(OH)D.

## Methods

### Participants

The Avon Longitudinal Study of Parents and Children (ALSPAC) is a prospective cohort that recruited pregnant women living in the South West of England and who had expected delivery dates between April 1991 and December 1992[22]. Children of non-white ethnic origin as identified using genetic data were excluded from all analyses. Offspring dental health data was recorded both via one or more parent-reported questionnaires at 38, 54, 65 and 77 months and a child self-reported questionnaire at 91 months. A random 10% sample of ALSPAC children born in the last 6 months of the study (June–December 1992) were invited to take part in a sub-study called Children in Focus[23]. At 31, 43 and 61 months of age, these children underwent a dental examination with decayed, missing and filled deciduous teeth (dmft) recorded[24]. The parents gave written informed consent to the participation of their children in the study. All aspects of the study were reviewed and approved by the ALSPAC Law and Ethics Committee, which is registered as an Institutional Review Board. (Approval was also obtained from the local research ethics committees, which are governed by the UK Department of Health.) Please note that the study website contains details of all the data that are available through a fully searchable data dictionary (http://www.bris.ac.uk/alspac/researchers/data-access/data-dictionary/).

### Genotyping

A total of 9,912 participants were genotyped using the Illumina HumanHap550 quad genome-wide SNP genotyping platform. Quality control assessment and imputation are described in S1 Text. When the dataset included singleton siblings born to the same mother, only the firstborn child was included in the analyses of children. Data for the SNPs is in the form of dosages that includes adjustment for imputation.

### Measurement of 25(OH)D concentrations

Measurement of 25(OH)D was done primarily on stored serum that had been collected at the 9 year follow-up assessment (mean age 9 years 9 months). If no samples were available from the assessment at age 9, samples from the 11-year assessment or the 7-year assessment were used. In total 6,504 children had a measure of serum 25(OH)D (4,441 from the 9 year assessment, 1,055 from the 7 year and 1,008 the 11 year assessment). The age range of the children with a vitamin D measure was 85 to 163 months.

After collection, samples were immediately spun, frozen, and stored at −80°C. Assays were performed after a maximum of 12 years in storage with no previous freeze-thaw cycles. The 25(OH)D_3_ and 25(OH)D_2_ concentrations were measured with HPLC tandem mass spectrometry using internal standard in a laboratory meeting the performance target set by the Vitamin D External Quality Assessment Scheme Advisory Panel for 25(OH)D assays. Interassay coefficients of variation for the assay were less than 10% across a working range of 2.5–624 nmol/L for both 25(OH)D_3_ and 25(OH)D_2_ [25]. Serum 25(OH)D concentrations were calculated as the sum of 25(OH)D_3_ and 25(OH)D_2_ and adjusted for seasonality.

### Outcome variables

Parental completed questionnaires obtained data on whether the child had ever had a filling or extraction; those who answered yes were defined has having experienced caries. It was not possible to obtain information on untreated decay from parental completed questionnaires. Child self reported questionnaires obtained data on if the child had ever had a filling, an extraction or had current untreated caries. Whether a child had received a GA for a dental extraction by 91 months was also recorded in both questionnaire types. The age of the child when the case-identifying questionnaire was completed was used as a measure of first caries experience. Questions from parental and child reported questionnaires are available in S2 Text. All participants had at least one parental reported and one child reported questionnaire, case status in either was used to identify a case. For the subgroup of children who were clinically assessed, evidence of untreated and treated caries were obtained and combined into a single variable representing examination assessed experience of caries. Severity of caries was recorded as dmft. For those with questionnaire and clinic assessment data (N = 624), caries experience was used to validate questionnaire data by identifying misclassified caries experience cases.

### Potential confounding factors

Consistent with other studies we have previously shown that season, age, sex, pubertal status, ethnicity, socioeconomic position, time spent outdoors and body mass index (BMI) are related to 25(OH)D concentrations[25]. For BMI a previous MR study has shown that it causally influences 25(OH)D concentration[21] and observational studies have reported associations between BMI and caries[26]. Of the remaining we considered that age, sex, ethnicity and socioeconomic position were also risk factors for dental caries and therefore confound the observational association. In this study we restricted analyses to participants who reported their ethnicity as “white”. Season of measurement of 25(OH)D was determined from the date at which the blood sample was obtained and values were adjusted for seasonality using a sine-cosine regression method as previously described[27]. Household occupational social class and maternal highest educational qualification were tested as measures of socioeconomic position with data on these obtained by questionnaire at recruitment of the mothers. Maternal educational qualification was found to have more of an effect than household occupational social class or both variables together, and therefore used alone in multivariable analyses. Sex was recorded at birth and age at the clinics when 25(OH)D was assessed. 25(OH)D and caries measurements were not recorded at the same time, the lag time between the last caries measure and the 25(OH)D measure was calculated.

### Statistical analysis

Multivariable regression analyses and MR instrumental variable analyses were employed to test observational and causal effects of the associations between 25(OH)D with dental caries outcomes. To explore the extent to which missing data might have biased our findings, we presented means (medians if the data were skewed) for continuous variables and proportions for categorical variables in the potentially available participants, participants involved in multivariate analysis and participants involved in MR analysis.

In both multivariable regression and MR instrumental variable analyses we examined the relationship of 25(OH)D and seasonally adjusted 25(OH)D as continuous variables, examining the association of each greater 10 nmol/L 25(OH)D with outcomes, as in previous studies. In the multivariable regression analyses we also examined associations with 25(OH)D categorized as: sufficient (>50 nmol/L), insufficient (30–49 nmol/L) and deficient (<30nmol/L)[28]. For caries onset we generated a binary variable that arbitrarily separates the lower tertile, early onset, from the upper 2 tertiles.

In the subset of participants with both clinical and questionnaire dental data we used a T-test to compare mean 25(OH)D between correctly and incorrectly classified cases.

We examined the associations of potential confounding factors with 25(OH)D categories and also with the SNP genotypes by presenting means or proportions by categories of 25(OH)D and genotype and using an F-test or χ^2^ to test for differences. Our a priori assumption was that 25(OH)D categories would be related to many of the confounders whereas the SNPs would not.

#### Multivariable regression

Logistic regression was used to examine the association of 25(OH)D (continuous or categorical variable) with caries experience, dental GA, caries onset and clinically measured caries experience. The clinical assessment severity data had a non-normal distribution. In STATA zero-inflated Poisson regression (‘zip’)[29] was used to assess the association of 25(OH)D with caries severity (dmft), using robust variance estimators (‘vce(robust)’) [30]. The Poisson regression coefficients were back transformed into dmft counts. In these analyses we present six models: unadjusted; 1—adjusted for season; 2 –adjusted for season and time lag between 25(OH)D and caries measure; 3 –adjusted for season, time lag sex and age; 4—adjusted for model 3 plus mothers highest education and 5—adjusted for model 3 plus BMI.

#### Instrumental variable analyses

SNP imputation quality was assessed using r^2^ from MACH imputation software. For MR analyses, the three SNP dosages were combined to produce a genetic risk score of between 0 and 6 weighted by the strength of the effect of each individual variant on 25(OH)D recorded in the discovery paper[19]. This instrument was assessed with respect to its association with potential confounders across quintiles of the genetic risk score.

Logistic structural mean models were used for MR analysis of caries experience, dental GA and caries onset[31]. The small sample size meant we did not examine effects on clinically recorded caries experience or severity (dmft) with the MR approach.

Considering the reported upper confidence interval of effect of vitamin D supplementation on caries (risk ratio = 0.63)[15] a sample size of 4155 would be required to detect a causal effect of 25(OH)D concentration on caries experience using MR with 90% power (α = 0.05)[32].

All analysis was performed using STATA 13 (Stata Corp, Texas, USA).

## Results

**Fig 1:**
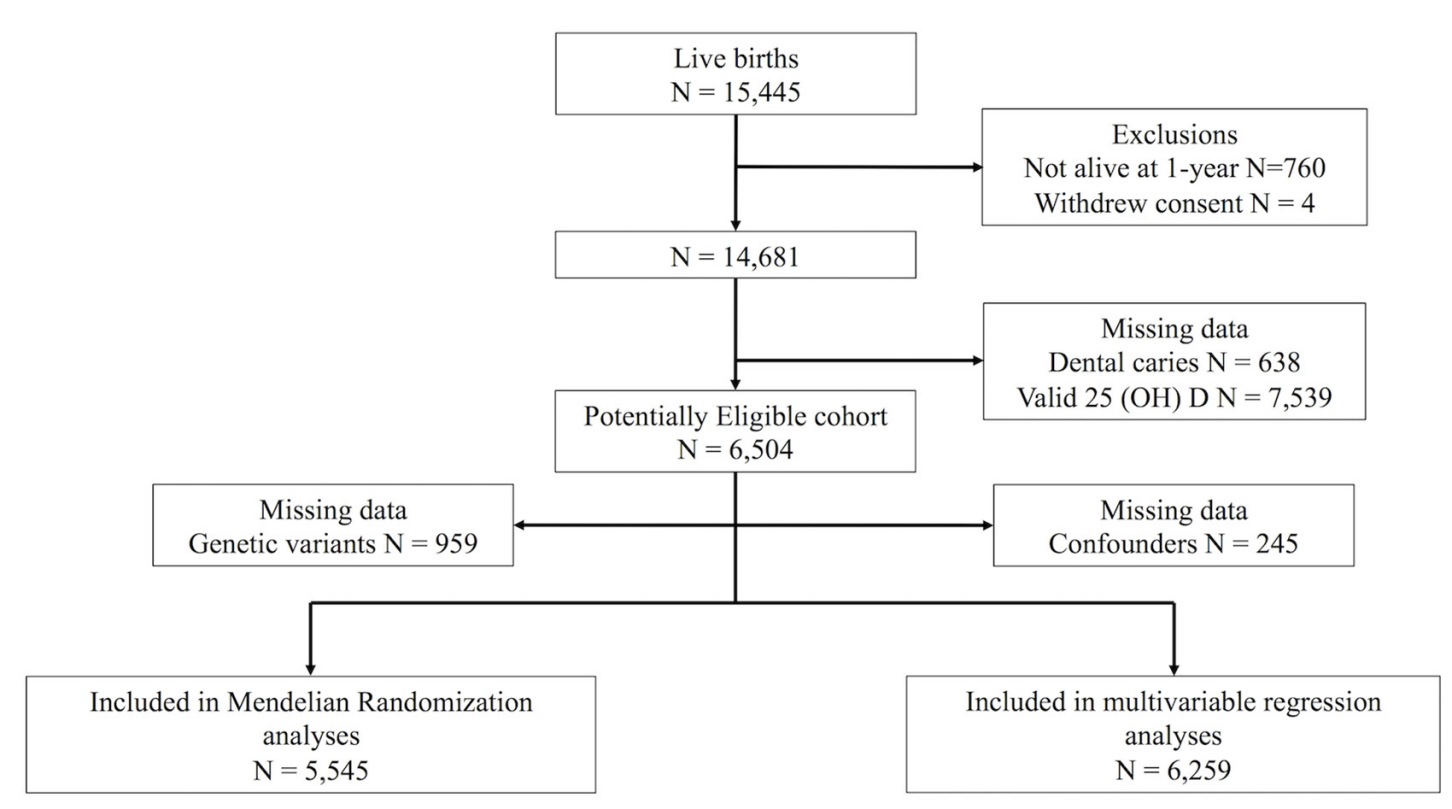
Study profile.

Fig 1 shows the flow of participants through the study and provides details of missing data. After excluding participants who died in utero or the first year of life and the small number who withdrew consent there were potentially 14,681 participants available for study. Of these 8,117 had no data on caries or 25(OH)D. Removing participants with missing confounder data left 6,259 for inclusion in the multivariable regression analyses. 959 participants were missing genetic variant data and therefore 5,545 were included in the MR analyses.

Mean age of blood sample for all participants with 25(OH)D assessment (n = 6,507) was 9 years 9 months with an overall median concentration of 60.9 nmol/L (IQR: 48.2, 75.1).

Amongst all 14,043 children with data on dental caries 3,839 (27.3%) had caries experience with a median age of onset of 77 months (IQR: 65, 91). Of the 7,045 with data on dental GA 716 (10.2%) had a dental GA by 91 months. The equivalent results for participants with complete data for multivariate analysis and MR analysis are shown in Table 1. Of the 916 children who received clinical dental examinations, modal dmft was 0 (range: 0, 13). In participants included in multivariate analysis (n = 6,259) caries experience was similar in females (37.0%) and males (36.0%) (*P* = 0.37) as were the median age of caries onset (females: 78 months (IQR:65, 91); males: 77 months (IQR: 65, 91)).

**Table 1:** Baseline characteristics of 25-hydroxyvitamin D, clinical outcomes and confounders in all potentially eligible participants and those with complete data for multivariate and MR analysis.

	All eligible participants		Multivariate analysis	MR analysis
	n = 14,681		n = 6,259	n = 5,545
Variable	n	Mean (SD) or N (%)	Mean (SD) or N (%)	Mean (SD) or N (%)
**Serum 25(OH)D (nmol/L)** ^a^	6,504	60.9 (48.4–75.0)	60.9 (48.4–74.9)	60.9 (48.4–74.9)
**Age at vitamin D measurement (months)**	6,504	117.3 (14.9)	117.3 (14.8)	118 (15)
**Female**	14,681	7,146 (48.7)	3,076 (49.2)	2,745 (49.5)
**Caries at 91 months**	14,043	3,839 (27.3)	2,285(36.5)	1933 (34.9)
*Age of caries onset* ^a^		*77 (65–91)*	*78 (65–91)*	*77 (65–91)*
*Early caries onset*		*1*,*285 (33*.*5)*	*726 (31*.*8)*	*614 (31*.*8)*
**Maternal highest educational qualification**	12,408			
CSE		2,503 (20.2)	827 (13.2)	739 (13.4)
Vocational		1,224 (9.86)	525 (45)	460 (8.3)
O Level		4,292 (34.6)	2,234 (35.7)	1,932 (34.9)
A Level		2,790 (22.5)	1,639 (26.2)	1,457 (26.4)
Degree		1,599 (12.9)	1,034 (16.5)	942 (17.0)
**Body mass index (kg/m** ^**2**^ **)** ^a^	9,264	17.1 (15.7–19.3)	16.9 (15.6–18.9)	16.9 (15.6–18.9)
**Dental GA at 91 months**	7,045	716 (10.2)	394 (8.3)^b^	332 (8.2)^c^

^a^ Median and interquartile range are displayed as variable is skewed

^b^ n = 4,742

^c^ n = 4,072

*Italics*—Caries cases only. MR = Mendelian randomization.

The latest clinical dental assessment was at 61 months, and allowed for assessment of agreement between caries experience from clinical assessment and questionnaire at 65 months (the nearest questionnaire to the last clinic assessment) (Table 2). In comparisons with clinically recorded dental caries data (taken as “gold standard”), 6% of questionnaire caries cases were false positives and 13% of questionnaire controls were false negatives. There was no substantial evidence of a difference in 25(OH)D concentration between correctly- (mean (SD) = 65.8 (22.4)) and incorrectly-classified (mean (SD) = 65.4 (21.8)) participants (*P* = 0.90).

**Table 2:** Cross-tabulation showing 25-hydroxyvitamin D by caries experience recorded at clinical examination (61 months) and by parent reported questionnaire (65 months).

Clinical data		Questionnaire data		
		No caries	Caries	Total
**No caries**	N	474	5	479
	Median (IQR) 25(OH)D	63.9 (52.7–76.4)	61.2 (55.9–82.4)	63.9 (52.7–76.4)
**Caries**	N	72	73	145
	Median (IQR) 25(OH)D	62.9 (51.2–72.3)	59.2 (49.9–76.9)	59.9 (50.7–75.6)
**Total**	N	546	78	624
	Median (IQR) 25(OH)D	63.5 (52.2–76.4)	59.5 (49.9–77.4)	62.9 (51.9–76.4)

### Associations of 25(OH)D and vitamin D SNPs with potential confounders

For sex, age, BMI and lag time there was strong evidence for association with 25(OH)D (Table 3). In contrast with this there was no evidence for association between measured confounders and genetic risk score for vitamin D (Table 4). No single SNP showed any association with any measured confounders (data not shown).

**Table 3:** Potential confounders across quintiles of 25-hydroxyvitamin D.

Quintiles of 25-hydroxyvitamin D							
	1 %	2 %	3 %	4 %	5 %	Total %	P-value
**Sex**							
Female	52.8	49.5	52.1	47.4	43.8	49.1	<0.001
**Highest qualification**							0.467
CSE	14.9	13.5	11.0	13.2	13.4	13.2	
Vocational	7.7	9.1	8.1	8.7	8.3	8.4	
O level	34.4	34.2	37.2	35.8	36.9	35.7	
A level	25.5	27.4	27.3	25.2	25.5	26.2	
Degree	17.6	15.8	16.3	17.1	15.8	16.5	
**Parental social class**							0.147
I	16.0	16.7	14.1	17.3	15.1	15.9	
II	43.1	42.9	47.1	44.4	46.6	44.8	
III (non-manual)	25.2	24.5	26.7	23.0	25.1	24.9	
III (manual)	11.5	11.4	8.6	10.7	9.6	10.4	
IV	3.4	4.1	3.2	4.0	3.1	3.6	
V	0.8	0.4	0.3	0.7	0.4	0.5	
	Mean (SD)	Mean (SD)	Mean (SD)	Mean (SD)	Mean (SD)	Mean (SD)	
**Age of 25(OH)D measure (months)**	119.9(13.5)	120.4(13.8)	119.1(13.6)	117.1(14.1)	110.1(16.3)	117.3(14.8)	<0.0001
	Median (IQR)	Median (IQR)	Median (IQR)	Median (IQR)	Median (IQR)	Median (IQR)	
**BMI (kg/m** ^**2**^ **)**	17.2(15.7–19.5)	17.2(15.7–19.4)	17.1(15.8–19.0)	16.8(15.6–18.7)	16.5(15.4–18.2)	16.9(15.6–18.9)	<0.0001
	Median (IQR)	Median (IQR)	Median (IQR)	Median (IQR)	Median (IQR)	Median (IQR)	
**Lag time (months)**	27(24–42)	27(24–44)	27(24–42)	26(23–39)	24(10–30)	26(23–40)	<0.0001
**N**	1,259	1,262	1,243	1,245	1,250	6,259	

**Table 4:** Potential confounders across quintiles of genetic score.

Quintiles of Genetic Risk Score							
	1 %	2 %	3 %	4 %	5 %	Total %	P-value
**Sex**							
Female	49.7	49.3	48.7	50.0	49.7	49.5	0.978
**Highest qualification**							0.102
CSE	14.3	12.7	12.3	13.2	13.9	13.4	
Vocational	10.0	6.8	8.8	8.6	6.7	8.3	
O level	34.2	35.0	33.0	35.2	37.4	34.9	
A level	25.3	28.3	28.0	26.7	24.1	26.3	
Degree	16.1	17.2	17.9	16.4	17.8	17.0	
**Parental social class**							0.261
I	14.9	17.5	17.6	14.8	16.6	16.2	
II	44.2	42.6	47.3	44.6	45.7	44.9	
III (non-manual)	25.2	25.1	22.7	23.9	24.4	24.3	
III (manual)	11.8	11.5	8.4	11.6	9.9	10.7	
IV	3.4	2.9	3.5	4.3	3.1	3.5	
V	0.6	0.5	0.4	0.7	0.3	0.5	
	Mean (SD)	Mean (SD)	Mean (SD)	Mean (SD)	Mean (SD)	Mean (SD)	
**Age of 25(OH)D measure (months)**	118.3(14.9)	117.8(14.9)	116.7(14.7)	117.6(14.2)	117.4(14.4)	117.6(14.6)	0.094
	Median (IQR)	Median (IQR)	Median (IQR)	Median (IQR)	Median (IQR)	Median (IQR)	
**BMI (kg/m** ^**2**^ **)**	17.0(15.6–19.1)	16.9(15.6–19.2)	16.8(15.6–18.7)	16.9(15.7–18.9)	17.1(15.7–19.1)	16.9(15.6–18.9)	0.608
	Median (IQR)	Median (IQR)	Median (IQR)	Median (IQR)	Median (IQR)	Median (IQR)	
**Lag time (months)**	27(23–42)	26(23–40)	26(23–38)	26(23–40)	26(23–41)	26(23–40)	0.999
**N**	1,364	871	1,096	1,113	1,101	5,545	

### Multivariable regression analyses

As a continuous variable 25(OH)D did not show convincing evidence of association with caries experience in unadjusted or confounder adjusted analyses (1.00 (95% CI: 0.97, 1.02) *P* = 0.79) (Table 5). When corrected for season and lag time (model 2) weak evidence for an association was found however this is lost with further adjustment for confounders. There was some evidence that higher 25(OH)D concentrations were related to a reduced odds of dental GA, with an odds ratio of 0.94 (95% CI: 0.89, 1.00; *P* = 0.05) per 10 nmol/L increase in 25(OH)D in the confounder adjusted model (Table 5). Among the 2,285 children who had experience of caries the age of onset appeared younger with higher 25(OH)D concentrations (0.93 (95% CI: 0.88, 0.98; *P* = 0.01). Among the 619 with clinic assessed caries, the OR for caries experience per 10 nmol/L increase in 25(OH)D was 0.93 (95% CI: 0.83, 1.04; *P* = 0.19) in fully adjusted models, although the confidence intervals were wide and included the null (Table 5). When caries severity from clinical assessment was analyzed the severity score was lower by 0.95 counts of dmft (95% CI: 0.88, 1.03; *P* = 0.22) in models adjusting for season, age and sex (S1 Table). The proportion of participants with caries was similar in participants with deficient and insufficient 25(OH)D when compared to those with sufficient 25(OH)D (S2 Table).

**Table 5:** Multivariate and MR analysis of caries experience, dental GA, caries onset and clinically recorded caries experience per 10 nmol/L increase in vitamin D.

	Caries experience (n = 6,259)		Dental GA (n = 4,737)		Early caries onset (n = 2,285)		Clinically recorded caries experience^$^ (n = 619)	
Model	OR (95% CI)	*P*-value	OR (95% CI)	*P*-value	OR (95% CI)	*P*-value	OR (95% CI)	*P*-value
Unadjusted	1.00 (0.97, 1.02)	0.70	0.95 (0.90, 0.99)	0.03	0.96 (0.93, 1.00)	0.08	0.94 (0.94, 1.03)	0.22
1	1.00 (0.97, 1.02)	0.73	0.94 (0.89, 1.00)	0.04	0.96 (0.91, 1.00)	0.07	0.93 (0.84, 1.04)	0.19
2	0.97 (0.94, 0.99)	0.02	0.94 (0.88, 1.00)	0.04	0.98 (0.93, 1.03)	0.36	0.93 (0.84, 1.04)	0.19
3	1.00 (0.97, 1.03)	0.95	0.95 (0.89, 1.00)	0.08	0.93 (0.88, 0.98)	0.01	0.93 (0.84, 1.03)	0.18
4	1.00 (0.97, 1.02)	0.75	0.94 (0.89, 1.00)	0.05	0.93 (0.88, 0.98)	0.01	0.93 (0.83, 1.04)	0.19
5	1.00 (0.97, 1.02)	0.79	0.94 (0.89, 1.00)	0.05	0.93 (0.88, 0.98)	0.01	0.93 (0.83, 1.04)	0.19
**n**	**5545**		**4072**		**1933**			
**MR**	0.93 (0.83, 1.05)	0.26	0.96 (0.75, 1.22)	0.72	1.09 (0.89, 1.34)	0.37		

Model 1 = seasonally adjusted, model 2 = model 1 plus lag time, model 3 = model 1 plus age, sex, model 3 = model 2 plus mothers highest educational level, model 4 = model 3 plus body mass index

^$^ Due to small sample size MR analysis was not performed for this outcome

MR = Mendelian randomization

### MR Instrumental variable analyses

After quality control assessment and imputation, genotypes at SNPs rs2282679, rs10741657 and rs7944926 were available for 8,358 participants. Imputation quality was high with r^2^ imputation quality scores greater than 0.98 for all three SNPs. The minor allele frequencies were 0.40, 0.23 and 0.29 for *CYP2R1*rs10741657, *DHCR7*rs7944926 and *GC*rs2282679, respectively.

**Fig 2:**
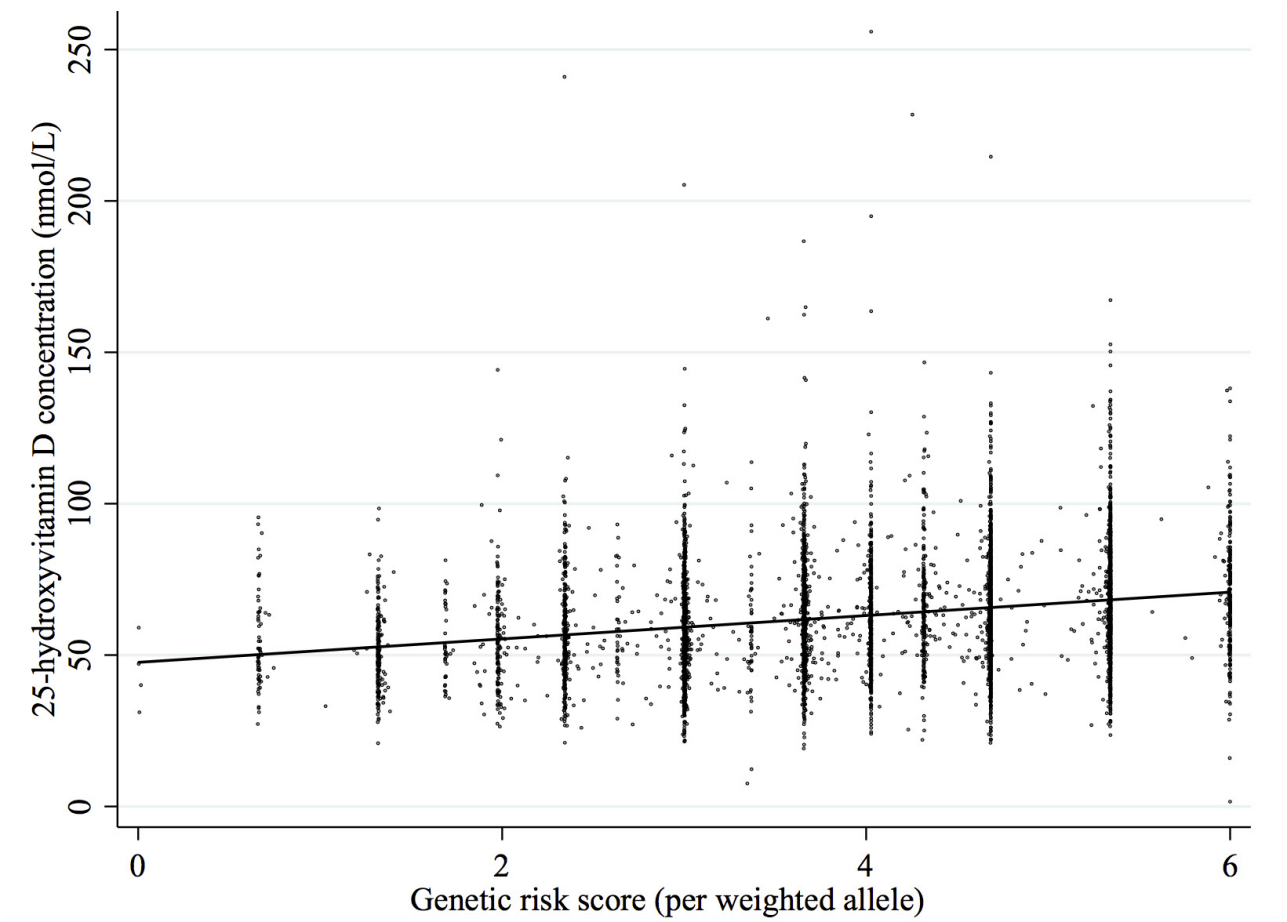
25-hydroxyvitamin D concentration across weighted genetic dose score.

Of the three gene variants *GC*rs2282679 showed the largest association with 25(OH)D, with concentration increasing by 6.13 nmol/L for each 25(OH)D increasing allele. Equivalent results for *CYP2R1*rs10741657 and *DHCR7*rs7944926 were 3.56 nmol/L and 2.15 nmol/L, respectively. Fig 2 shows the effect of the relationship of the allele score with 25(OH)D concentration. For each additional 25(OH)D weighted allele, 25(OH)D concentrations increased by 3.87 nmol/L. This genetic risk score explained 5.9% of the variation in 25(OH)D and the F-statistic for this association was 348.5.

In each MR analyses there was no convincing evidence of a causal association between increased 25(OH)D and the odds of caries experience (OR per 10 nmol/L increase in 25(OH)D 0.93 (95% CI: 0.83, 1.05; *P* = 0.26)) (Table 5). Estimates for dental GA and early onset caries (in those with caries) for the same change in 25(OH)D were OR 0.96 (95% CI: 0.75, 1.22; *P* = 0.72) and OR 1.09 (95% CI: 0.89, 1.34; *P* = 0.37) (Table 5).

## Discussion

Existing literature has shown that vitamin D exposure in early life may play a role in caries prevention[15] However, in work here where 25(OH)D is available there was little evidence for an observational associations with caries experience. There was evidence of an association between 25(OH)D concentration with GA experience and earlier onset of caries—both of which could be seen as a proxy for more severe disease.

Within this population-based sample, the previously reported relationships between 25(OH)D and *CYP2R1*, *DHCR7* and *GC* were replicated[18, 19]. When combined these SNPs were confirmed as a strong genetic instrument for 25(OH)D, with per allele changes of a clinically relevant magnitude. This genetic instrument showed no association with factors potentially confounding the vitamin D—dental caries relationship. Therefore, in the absence of coordinated vitamin D and outcome data this genetic instrument was used to model a life-course difference in vitamin D exposure for use in MR analyses.

MR analysis has not given substantive evidence for an association between caries experience and vitamin D. This is in contrast to a meta-analysis which reports on 14 controlled clinical trials assessing vitamin D_3_ as a supplement, compared to no supplement as a control[15]. They report a 49% (95% CI: 35, 60, *P*<0.0001) decrease in the relative rate of caries. This meta-analysis reports the relative rate of caries incidence, calculated by dividing the risk of caries by the length of the study, and not the odds of having caries experience, as done here, therefore comparison is difficult. Evidence here also sits in the context of recent work suggesting a potential role for vitamin D receptor in adult dental caries. The findings in this work were not replicated in the ALSPAC study (data not shown)[7].

Observationally, participants with higher serum 25(OH)D had reduced odds of receiving a GA for dental extractions. It is of note that an observational association exists with GA experience but not with caries experience despite the former usually considered a proxy for the latter. This may be because GAs are a memorable event and therefore more likely to be reported accurately. It has also been reported that GA for dental extractions is much more common in lower socio-economic groups[5] and despite adjusting, may be additionally confounding the link between vitamin D and GA experience. Care is needed when interpreting this result as it presumes vitamin D concentrations are consistent throughout childhood, although evidence remains after correcting for the time lag between caries and vitamin D measurement. The MR analysis is underpowered with wide confidence intervals meaning inference is limited. Interestingly, early caries onset MR point estimates, are in the opposite direction to that of the other two caries outcomes, although a larger sample size would be required to clarify these results.

### Limitations

Using questionnaire data for outcomes has several weaknesses and is likely to underreport caries compared to the “gold-standard” clinical examination. In this study we were able to internally validate the questionnaire findings in a sample of participants who also had clinical dental examinations (Table 2). 13% of questionnaire reported controls had clinically recorded caries experience and 6% of cases has no clinically recorded caries. There was no substantive evidence of vitamin D being different across mis-allocated groups with there being similar 25(OH)D concentrations across data origins. There was also no association between the questionnaire type that identified a participant as a case (parent or self report) and 25(OH)D (S3 Table). The unresolved problem with the questionnaire data is under-identification of untreated disease. This was only asked in the child reported questionnaire at 91 months but even so, is likely to have poor sensitivity and therefore be responsible for the majority of the underreporting.

The binary caries outcome does not account for disease severity, although dmft (which showed comparable results) provides a measure for this. Additionally, the caries onset analyses relied on the dates of questionnaire completion, rather than the actual age at which caries was diagnosed, introducing error. The time difference between vitamin D and outcome measures is a limitation in the observational part of this study. In the observational analysis we attempt to correct for this by including lag time between caries and 25(OH)D measures as a confounder. In the MR analysis we address this by using a life-course genetic instrument for vitamin D, however this does assume the effects of the genes used to instrument vitamin D are stable throughout childhood.

This study is large in comparison to previous observational studies assessing the association between vitamin D and caries, however it has relatively low power to detect associations between vitamin D and dental caries smaller than those previously reported in observational studies. Given the prevalence of caries in this population and the strength of the genetic instrument this study had sufficient size to detect an effect similar to that reported by Hujoel[15]. Considering the observational results in this study it is likely the effect size in this population is smaller, resulting in the MR analysis being underpowered. This genetic instrument has favorable properties when investigating the association between 25(OH)D and caries, but there is currently no conclusive evidence of a causal effect. Further effort is required in the field of oral health epidemiology to collect good quality clinically recorded outcomes alongside genetic data. These improvements in sample size and measurement will allow more precise estimates of causal association in future work.

## Conclusion

With the use of a genetic instrument, a life-course 25(OH)D exposure model was used to test associations between vitamin D and dental caries. In contrast to a recent meta-analysis[15] we found little evidence of an observational association between 25(OH)D and caries experience and severity and only weak evidence for caries onset and receiving a dental GA. Genetic instruments for 25(OH)D appeared not to be confounded and were strong, though MR estimates spanned the null for all outcomes. Genetic study designs of this type show promise for the future and efforts should be made to identify other potential instrumental variables for use in oral health research.

## Supporting Information

S1 Text Avon Longitudinal Study of Parents and Children (ALSPAC) GWAS. (DOCX)

S2 Text Parental Reported and Child Self Reported Questionnaire Questions. (DOCX)

S1 Table Multivariate analysis of clinically recorded caries experience and dmft per 10 nmol/L increase in vitamin D. (DOCX)

S2 Table Caries Experience Across Vitamin D Sufficiency Group. (DOCX)

S3 Table 25(OH)D and Genetic Risk Score by Reporter of Case Status. (DOCX)
