# Supplementary material for: Re-Examining the Association between Vitamin D and Childhood Caries
Source: PLoS One. 2015 Dec 21;10(12):e0143769. doi: 10.1371/journal.pone.0143769 (PMC4686942; doi:10.1371/journal.pone.0143769)
Supplement: S1 Text — (DOCX) [file pone.0143769.s001.docx]

**S1 Text. Avon Longitudinal Study of Parents and Children (ALSPAC) GWAS.**

A total of 9,912 participants were genotyped using the Illumina HumanHap550 quad genome-wide SNP genotyping platform by Sample Logistics and Genotyping Facilities at the Wellcome Trust Sanger Institute and LabCorp (Laboratory Corportation of America) supported by 23andMe. Individuals were excluded from further analysis on the basis of having incorrect sex assignments; extreme heterozygosity (<0.320 and >0.345 for the Sanger data and <0.310 and >0.330 for the LabCorp data); high levels of individual missingness (>3%); evidence of cryptic relatedness (>10% IBD) and being of non-European ancestry (as detected by a multidimensional scaling analysis seeded with HapMap 2 individuals). EIGENSTRAT analysis revealed no additional obvious population stratification and genome-wide analyses with other phenotypes indicate a low lambda. The resulting data set consisted of 8,358 individuals. SNPs with a minor allele frequency of <1% and call rate of <95% were removed. Only SNPs which passed an exact test of Hardy–Weinberg equilibrium (p >5 × 10^-7^) were considered for analysis. Known autosomal variants were imputed with MACH 1.0.16 Markov Chain Haplotyping software (1, 2), using CEPH individuals from phase 2 of the HapMap project (HG18) as a reference set (release 22).

**References**

1. Li Y, Willer C, Sanna S, Abecasis G. Genotype imputation. Annual review of genomics and human genetics 2009;10:387-406. doi: 10.1146/annurev.genom.9.081307.164242.

2. Li Y, Willer CJ, Ding J, Scheet P, Abecasis GR. MaCH: using sequence and genotype data to estimate haplotypes and unobserved genotypes. Genetic epidemiology 2010;34(8):816-34. doi: 10.1002/gepi.20533.
