# Supplementary material for: Re-Examining the Association between Vitamin D and Childhood Caries
Source: PLoS One. 2015 Dec 21;10(12):e0143769. doi: 10.1371/journal.pone.0143769 (PMC4686942; doi:10.1371/journal.pone.0143769)
Supplement: S2 Text — (DOCX) [file pone.0143769.s002.docx]

**S2 Text. Parental and child reported questions.**

**Parent reported questions in questionnaires at 38, 54, 65 and 77 months:**

- Has he/she visited the dentist since [time since last questionnaire]?
  - Yes for treatment
    - A filling? = case
    - A tooth taken out? = case
      - How many? Not used
      - Did they have a general anaesthetic for this? = GA case
    - Other dental treatment?
  - Yes, for inspection only Yes = control
  - No, not at all = control

**Child self reported questions in questionnaires at 91 months:**

- Have you ever had a filling?
  - Yes = case
  - No = control
- Have you ever been given something to make your mouth go numb (sleepy, frozen, dead)?
  - Yes
    - What did you have done to your teeth at that time?
      - A filling = case
      - Tooth pulled out = case
      - something else (please say what) ........ Not used
  - No = control
- Have you ever been given something to make you go to sleep (general anaesthetic) before the dentist did something to your teeth?
  - Yes
    - What did you have done to your teeth at that time?
      - Tooth pulled out = case, GA case
      - something else (please say what) … Not used
  - No = control
- Have you ever had a magic wind mixture that you breathe through a special nose-piece which makes you feel brave but lets you stay awake (sedation)?
  - Yes
    - What did you have done to your teeth at that time?
      - A filling = case
      - Tooth pulled out = case
      - something else (please say what) ........ Not used
  - No = control
- Please look in the mirror or get someone else to help.
  - How many fillings are there in your mouth? (don’t forget the front teeth!). answer > 0 = case
  - Looking in the mirror and feeling with your tongue:
    - How many teeth can you see or feel which have a hole in them? Answer >0 = case
