# Supplementary material for: Re-Examining the Association between Vitamin D and Childhood Caries
Source: PLoS One. 2015 Dec 21;10(12):e0143769. doi: 10.1371/journal.pone.0143769 (PMC4686942; doi:10.1371/journal.pone.0143769)
Supplement: S1 Table — (DOCX) [file pone.0143769.s003.docx]

**S1 Table. Multivariate analysis of clinically recorded dmft**

**per 10 nmol/L increase in 25(OH)D.**

| Change in dmft count per 10 nmol/L increase in 25(OH)D (n=619) | | |
| --- | --- | --- |
| Model | dmft (95% CI) | P-value |
| Unadjusted | 0.95 (0.90, 1.02) | 0.15 |
| Model 1 | 0.96 (0.89, 1.03) | 0.29 |
| Model 2 | 0.95 (0.88, 1.03) | 0.22 |
| Model 1 = seasonally adjusted, model 2 = model 1 plus age, sex | | |
