# Supplementary material for: Re-Examining the Association between Vitamin D and Childhood Caries
Source: PLoS One. 2015 Dec 21;10(12):e0143769. doi: 10.1371/journal.pone.0143769 (PMC4686942; doi:10.1371/journal.pone.0143769)
Supplement: S2 Table — (DOCX) [file pone.0143769.s004.docx]

**S2 Table. Caries Experience Across Vitamin D Sufficiency Groups**

|  | Deficient | Insufficient | Sufficient | Total |
| --- | --- | --- | --- | --- |
| No caries (%) | 52 (61.2) | 1123 (64.6) | 3030 (64.8) | 4205 (64.7) |
| Caries (%) | 33 (38.8) | 616 (35.4) | 1649 (35.2) | 2298 (35.3) |
| Total (%) | 85 (100.0) | 1739 (100.0) | 4679 (100.0) | 6503 (100.0) |
| Pearson χ2= 0.476 p=0.788 | | | | |
