# Supplementary material for: Re-Examining the Association between Vitamin D and Childhood Caries
Source: PLoS One. 2015 Dec 21;10(12):e0143769. doi: 10.1371/journal.pone.0143769 (PMC4686942; doi:10.1371/journal.pone.0143769)
Supplement: S3 Table — (DOCX) [file pone.0143769.s005.docx]

**S3 Table. 25(OH)D and Genetic Risk Score by Reporter of Case Status.**

25(OH)D summary by case status reporter.

| Case status report | n | Mean (SD) | Min | Max |
| --- | --- | --- | --- | --- |
| Parental | 1322 | 63.0 (22.6) | 16.7 | 201.2 |
| Child | 976 | 64.4 (21.8) | 9.7 | 175.2 |

T-test t= -1.47 (df=2296), p = 0.14

Genetic Risk Score by case status reporter

| Case status report | n | Mean (SD) | Min | Max |
| --- | --- | --- | --- | --- |
| Parental | 1441 | 3.95 (1.21) | 0.66 | 6.00 |
| Child | 1008 | 3.89 (1.23) | 0.66 | 6.00 |

T-test t= 1.18 (df=2447), p = 0.24
